# Supplementary material for: TRIM25 predominately associates with anti-viral stress granules
Source: Nat Commun. 2024 May 15;15:4127. doi: 10.1038/s41467-024-48596-4 (PMC11096359; doi:10.1038/s41467-024-48596-4)
Supplement: Supplementary file 1 — Supplementary Information [file 41467_2024_48596_MOESM1_ESM.pdf]

Supplementary Figures

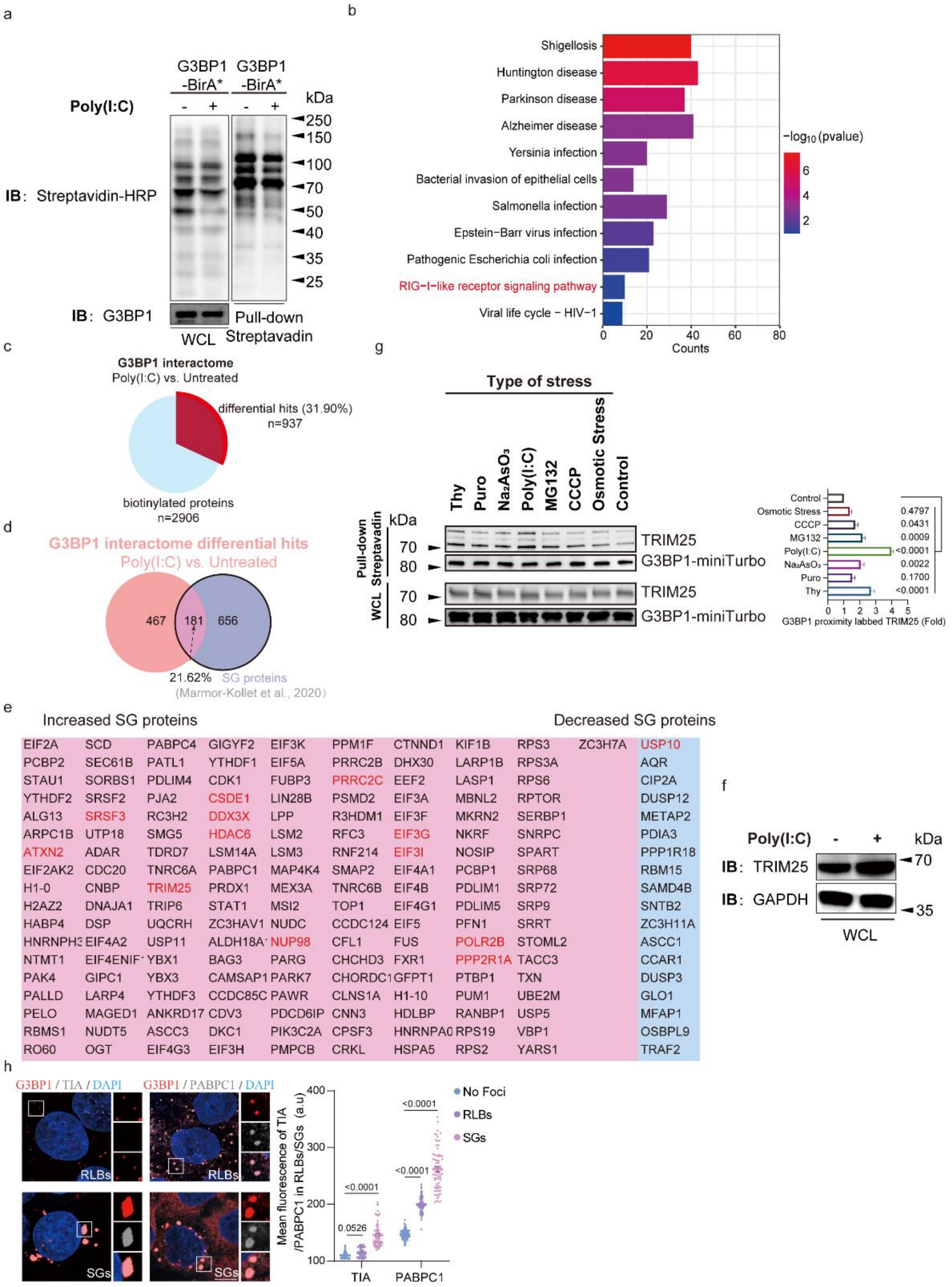

Supplementary Figure 1 | Proteomic analysis of Poly(I:C)-induced stress granules.

a) Immunoblotting analysis of proximity labeling using G3BP1-BirA* with or without poly(I:C) treatment in HEK293T cells. **b)** Pie chart showing the 937 differential hits out of 2906 biotinylated proteins in the G3BP1-asociated proteins. **c)** Representative enrichment score plot genes enriched in the RIG-I-like receptor signaling pathway, and viral or bacterial invasion KEGG pathway. **d)** Venn diagram showing a significant overlap between the G3BP1 interactome differential hits and the previously identified SG proteins. **e)** List of differential SG proteins identified in the G3BP1 interactome differential hits. SG core proteins are marked in red. **f)** The expression of TRIM25 in poly(I:C)-treated HEK293T, determined by immunoblotting. **g)** Immunoblotting analysis of streptavidin pull-down assay in HEK293T cells (left). **h)** Representative fluorescence microscopy images showing the colocalization of TIA or PABPC1 with RLBs (top left) or SGs (bottom left) in HeLa cells upon poly(I:C) stimulation. Statistical analysis of mean fluorescence intensity of TIA and PABPC1 in RLBs and SGs. For RLBs: TIA, n = 100; PABPC1, n = 100. For No Foci: TIA, n = 50; PABPC1, n = 98. For SGs: TIA, n = 71; PABPC1, n = 69; n represents the number of droplets. All data are representative of at least three independent experiments (**g, f, h**). Scale bars, 10 μm (**h**). (a.u.= arbitrary units.) Mean ± s.e.m., statistical analysis was performed using one-way ANOVA (**g**) or two-way ANOVA (**h**)

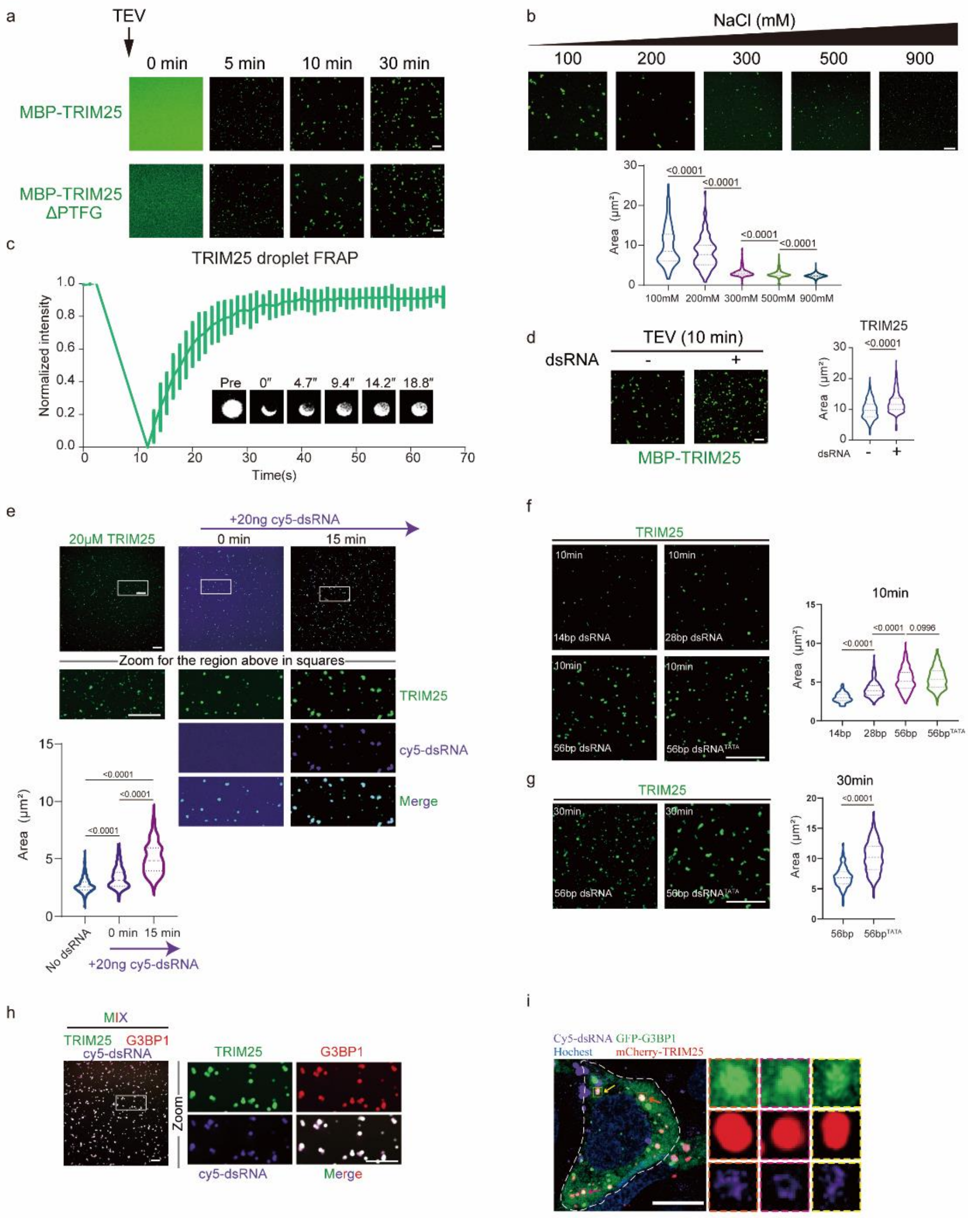

Supplementary Figure 2 | TRIM25 undergoes LLPS *in vitro*, and is further stimulated by dsRNA.

a) Time-dependent LLPS of MBP-TRIM25 WT and MBP-TRIM25 ΔPTFG. MBP-TRIM25 (50 μM) and 56bp-dsRNA (10 ng/μl) were mixed in a solution containing 20 mM Tris-HCl, pH 7.5, 150 mM NaCl, 8% v/v Ficoll, and TEV protease was added at 0 min to cleave the MBP tag. **b)** Salt-

concentration dependence of 50 μM TRIM25 LLPS. Quantitative analyses of TRIM25 droplet areas at different salt concentrations in b. 100 mM, n = 221; 200 mM, n = 126; 300 mM, n = 651; 500 mM, n = 862; 900 mM, n = 2718, n represents the number of droplets. **c)** FRAP analysis of TRIM25 droplets formed by 50 μM purified protein. Top: time-lapse images from the FRAP experiment. Bottom: fluorescence recovery curve after photobleaching inside the droplet over time. **d)** TRIM25 LLPS is promoted by dsRNA. MBP-TRIM25 (50 μM) and the 56bp-dsRNA (10 ng/μl) were mixed, and TEV was added at 0 min to cleave the MBP tag. **e)** cy5-dsRNA co-localizes with TRIM25. TRIM25 droplets were formed in a solution containing 20 mM Tris-HCl, pH 7.5, 150 mM NaCl, 8% v/v Ficoll. After formation of TRIM25 droplets, cy5-dsRNA (final concentration 2 ng/μl) was added, and TRIM25/cy5-dsRNA droplets were analyzed at different times. **f-g)** MBP-TRIM25 (20 μM) and dsRNA (10 ng/μl) were used, and TEV was added at 0 min to cleave the MBP tags. **h)** Cy5-dsRNA co-localizes with the TRIM25-G3BP1 co-condensate foci *in vitro*. MBP-TRIM25 (10 μM), MBP-G3BP1 (10 μM) and cy5-dsRNA (final concentration 2 ng/μl) were mixed, and TEV protease was added to initiate LLPS. **i)** Representative fluorescence microscopy images showing co-localization of Cy5-dsRNA with the co-condensate of TRIM25 and G3BP1 *in vivo*. HeLa cells were first transfected plasmids encoding mCherry-TRIM25 and GFP-G3BP1, and then 2 μg/ml cy5-dsRNA. Images were taken 4h after the cy5-dsRNA transfection. All data are representative of at least three independent experiments (**a-i**). Scale bars, 10 μm (**a, b, d, e, f, g, h, i**). Mean ± s.e.m., statistical analysis was performed using two-tailed Student's t-test (**d, j**) or one-way ANOVA (**b, e, f**).

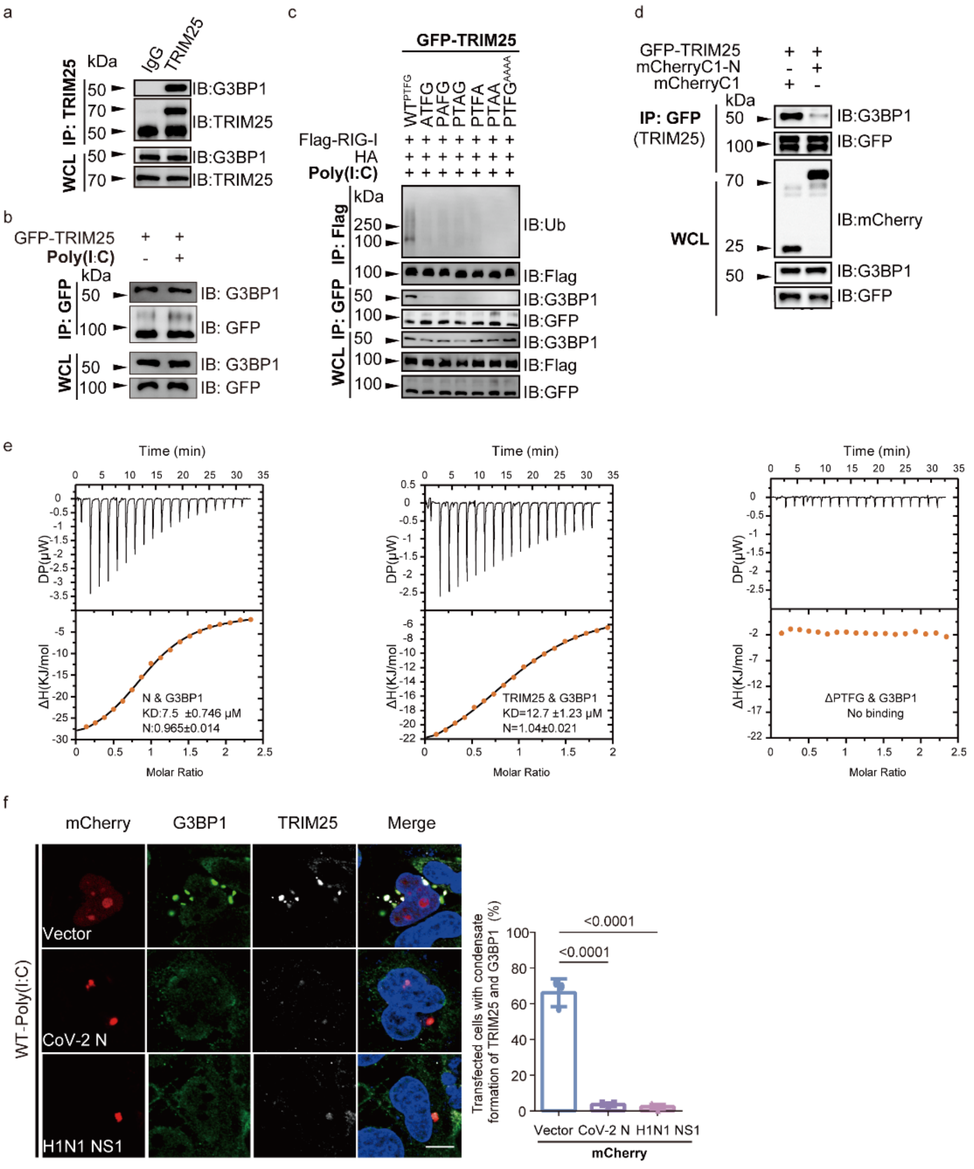

Supplementary Figure 3 | SARS-CoV-2 N protein inhibits the interaction and co-condensation between TRIM25 and G3BP1.

a) Co-precipitation of endogenous G3BP1 and TRIM25 from HEK293T cells. **b)** Immunoblotting analysis of HEK293T cells treated with or without poly(I:C). Cells transfected with GFP- TRIM25 were treated with or without poly(I:C) for 4 hours. Cell lysate was then collected and subjected to immunoprecipitation with GFP beads. **c)** Mutations in any amino acid of “PTFG” disrupt the TRIM25-G3BP1 binding and ubiquitination of RIG-I. HEK293T cells were co-transfected with

indicated constructs for 24 hours. Cells were collected and subjected to immunoprecipitation with GFP or Flag beads, and the immunoprecipitates were then detected with antibodies against GFP, Flag, ub, and G3BP1. GFP-IP assessed the TRIM25-G3BP1 binding; Flag-IP assessed the ubiquitination of RIG-I. **d)** SARS-CoV-2 N protein competitively inhibits TRIM25 binding to G3BP1. HEK293T cells were co-transfected with indicated constructs for 24 hours. Cells were collected and subjected to immunoprecipitation with GFP beads, and the immunoprecipitates were immunoblotted with antibodies against mCherry, GFP, and G3BP1. **e)** Isothermal titration calorimetry of N protein peptide or TRIM25 peptide (WT and ΔPTFG) titrated into purified G3BP1 protein in a buffer containing 100mM Tris pH 8.0, 500 mM NaCl, 5% v/v Glycerol at 25 ℃. Top and bottom panels show raw and integrated heat from injections, respectively. The black curve in the bottom panel represents a fit of the integrated data to a single-site binding model. **f)** Overexpression of SARS-CoV-2 N protein and H1N1 NS1 protein inhibited the formation of TRIM25 puncta and SGs in cells. All data are representative of at least three independent experiments (**a-f**). Scale bars, 10 μm (**f**). Mean ± s.d., statistical analysis was performed using one-way ANOVA (f).

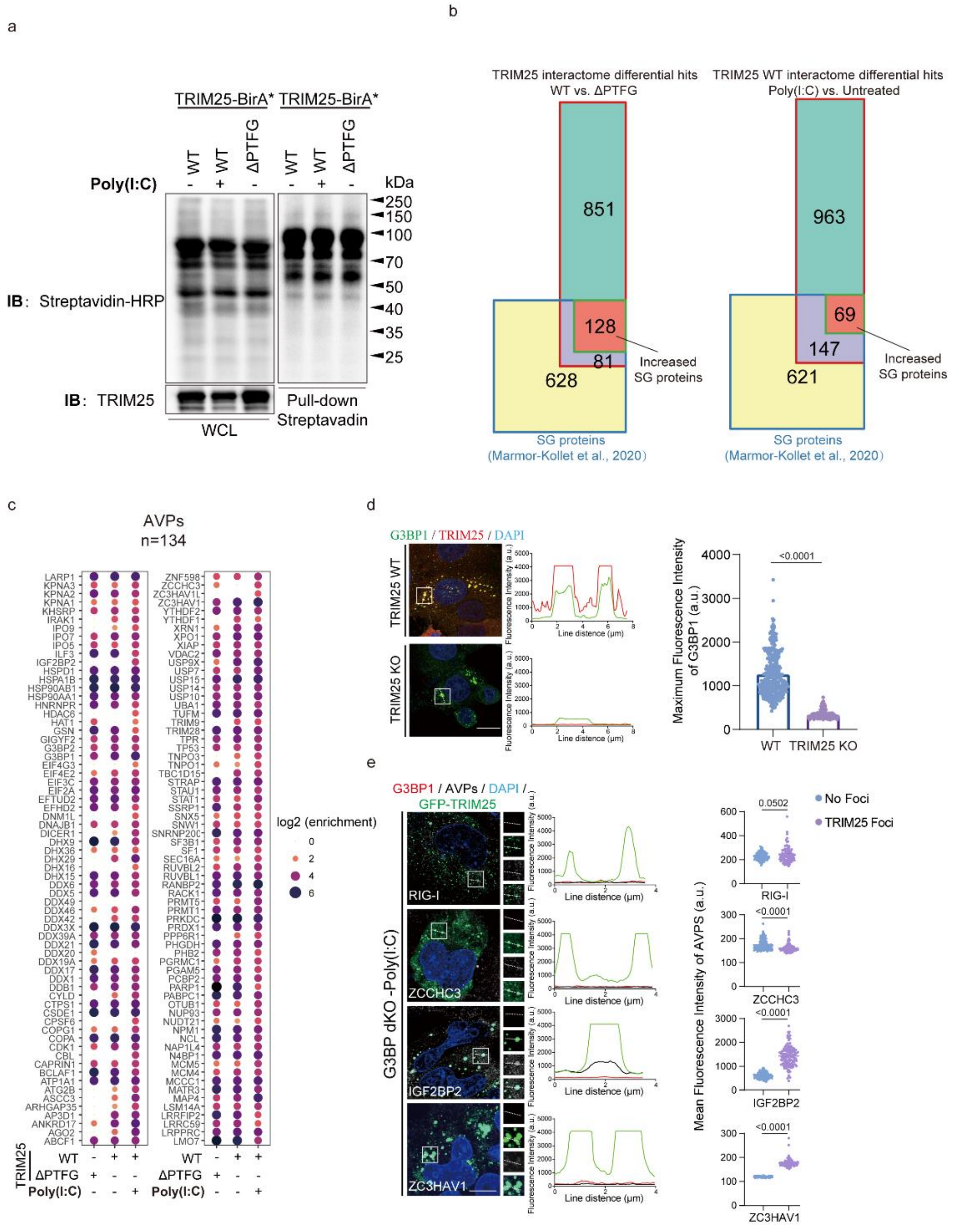

Supplementary Figure 4 | Co-condensation with G3BP1 facilitates TRIM25 to access SG-localized AVPs.

a) Immunoblotting analysis of proximity labeling using TRIM25 WT or ΔPTFG without poly(I:C) treatment, or TRIM25 WT with poly(I:C) treatment in HEK293T cells. **b)** Venn diagram showing significant overlap between TRIM25 interactome differential hits and previously identified SG proteins. **c)** Manually curated a list of 134 annotated Anti-Viral Proteins (AVPs) using MS/MS count>

9 criteria in the mass spectrometry data. ProHits-viz web tool was used to generate dot plot displaying prey abundance across baits and prey confidence. **d)** Representative images of TRIM25 WT and TRIM25 KO HeLa cells. The cells were treated with poly(I:C), then stained with antibodies against G3BP1 and TRIM25, finally stained with DAPI. Line scans show the related intensity profiles of TRIM25 and G3BP1. **e)** Representative images of AVPs subcellular localization in poly(I:C)-treated G3BP dKO HeLa cells expressing GFP-TRIM25. DAPI staining was shown in blue. Line scans show the related intensity profiles of TRIM25, G3BP1 and AVPs. Data are representative of at least three independent experiments (**d, e**). Scale bars (**d, e**), 10 μm. (a.u.= arbitrary units.) Mean ± s.e.m., statistical analysis was performed using two-tailed Student's t-test (**d, e**).

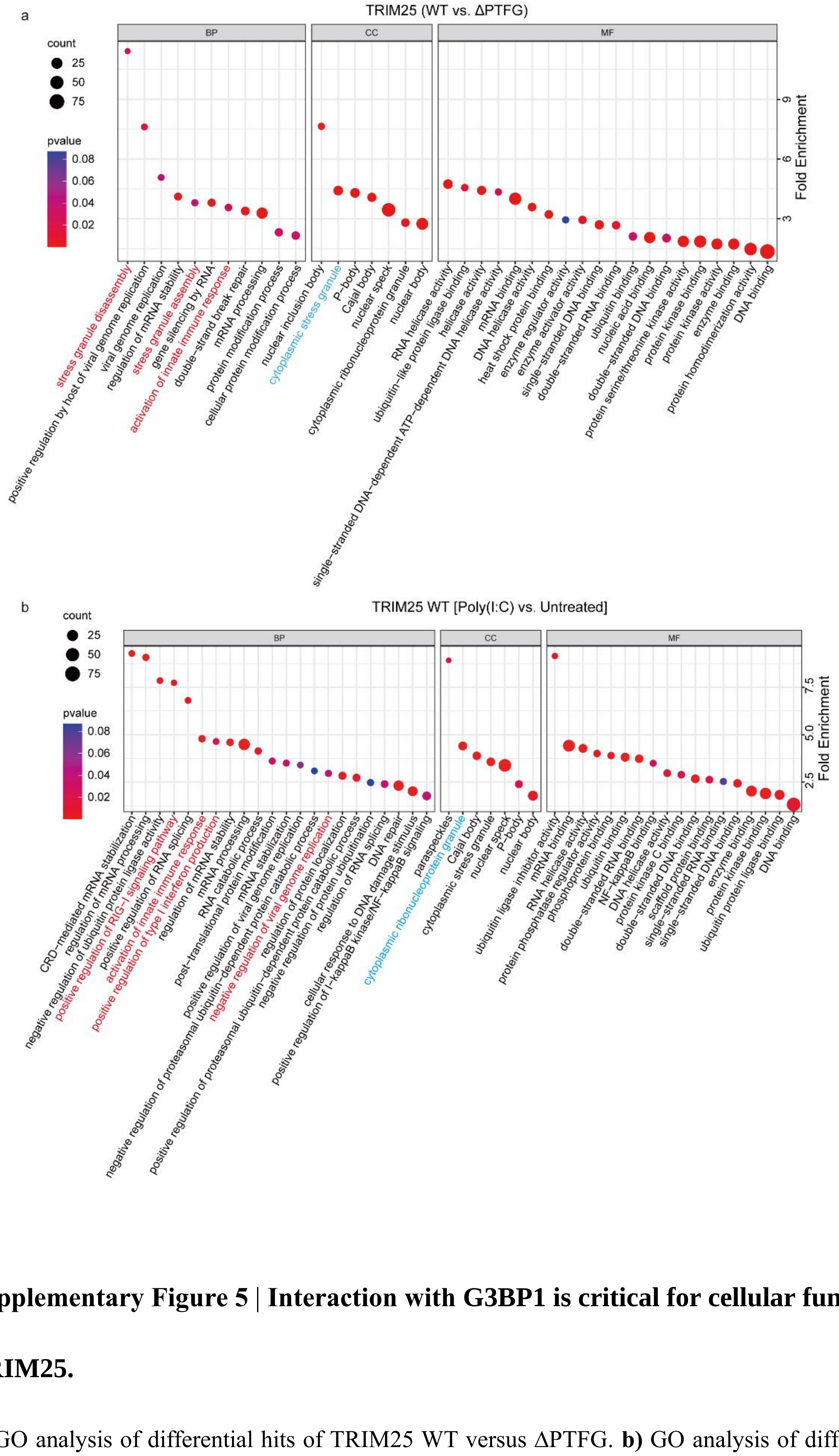

Supplementary Figure 5 | Interaction with G3BP1 is critical for cellular functions TRIM25.

a) GO analysis of differential hits of TRIM25 WT versus ΔPTFG. **b)** GO analysis of differential hits of TRIM25 WT poly(I:C) treatment versus untreated.

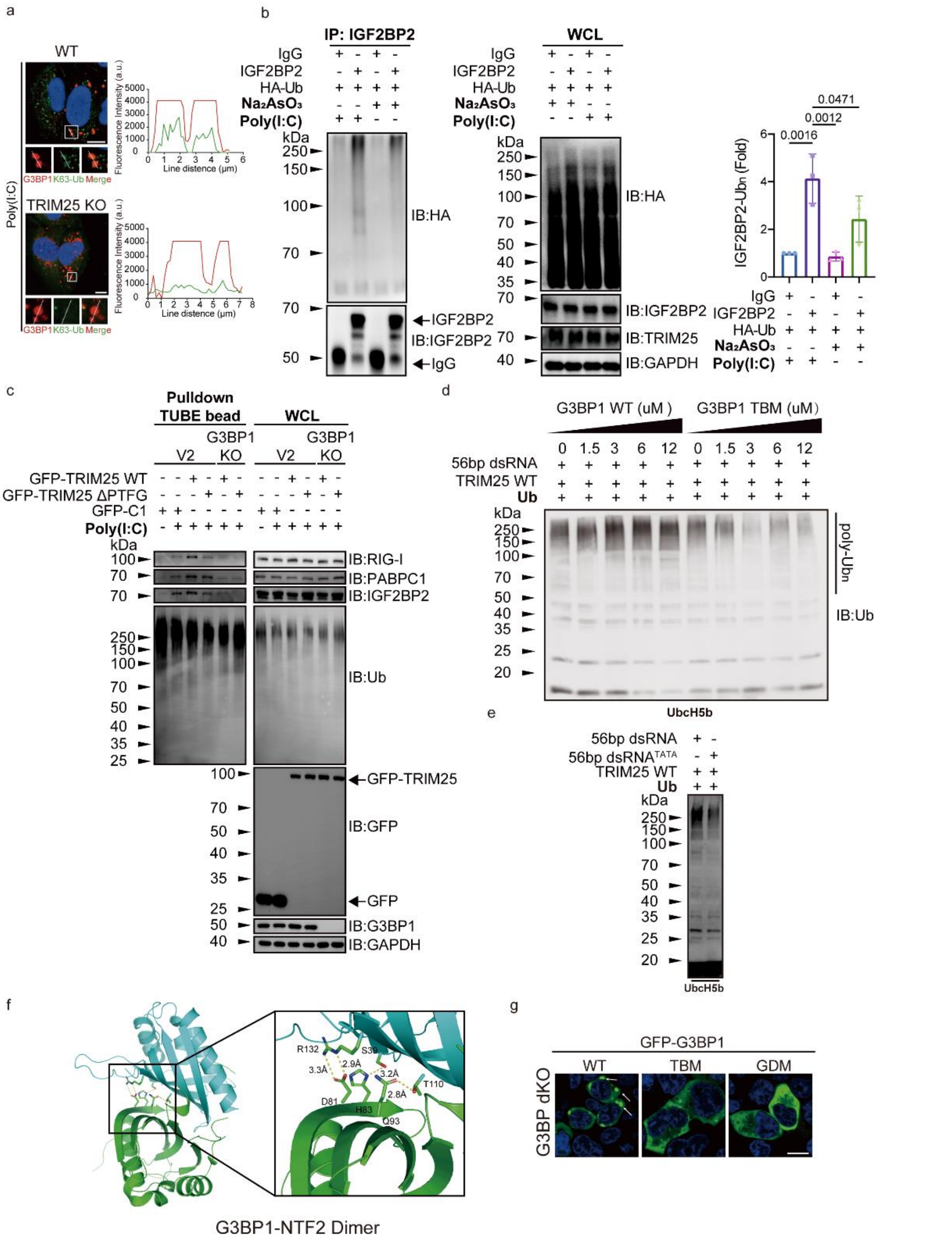

Supplementary Figure 6 | Co-condensation with G3BP1 enhances ubiquitination activities of TRIM25 toward various substrates *in vitro*.

a) WT and TRIM25 KO HepG2 cells were treated with poly(I:C), fixed, and then immunostained with antibodies against G3BP1 and K63-Ub. Line scan analysis was used to show colocalization. **b)**

WT or TRIM25 KO HepG2 cells were transfected with the HA-Ub and treated with poly(I:C). The cell lysates were subjected to immunoprecipitation with an IGF2BP2 antibody, followed by immunoblotting with indicated antibodies. **c)** Immunoblots of TUBE-captured cell extracts showing levels of ubiquitinated RIG-I, PABPC1, and IGF2BP2 in WT or G3BP1 KO HEK293T cells. The cells were transfected with TRIM25 WT or ΔPTFG, and then treated with poly(I:C). **d)** *In vitro* ubiquitination analysis. Assays contained purified E1, E2 (UbcH5b), E3 (TRIM25), 56-bp dsRNA, Ub, and various concentrations of G3BP1 WT or G3BP1 TBM. Poly-Ub chain was analyzed by anti-Ub blot. **e)** *In vitro* ubiquitination analysis. Assays contained purified E1, E2 (UbcH5b), E3 (TRIM25), Ub, and dsRNA (56bp-dsRNA or 56bp-dsRNA^{TATA}). Poly-Ub chain was analyzed by anti-Ub blot. **f)** The G3BP1 NTF2 dimer interface (PDB:4FCJ). Residues at the dimer interface are shown as sticks and labeled. Dotted lines: hydrogen bonds. **g)** G3BP dKO HEK293T cells were transfected with the indicated G3BP1 mutant plasmids. Representative microscopy images to show the formation of G3BP1 droplets. White arrows indicate the G3BP1 droplets. All data are representative of at least three independent experiments (**a-e, g**). Scale bars, 10 μm (**a, g**). (a.u.= arbitrary units.) Mean ± s.d., statistical analysis was performed using one-way ANOVA (**b**).

Supplementary Table S1: Sequence of qPCR primers

Target	Forward (5' - 3')	Reverse (5' - 3')
GAPDH	AGAAGGCTGGGGCTCATTTG	AGGGGCCATCCACAGTCTTC
IFNα	GCCTCGCCCTTTGCTTTACT	CTGTGGGTCTCAGGGAGATCA
IFNβ	GATTCATCTAGCACTGGCTGG	CTTCAGGTAATGCAGAATCC
IFNγ	CGCCTTGGAAGAGTCACTCA	GAAGCCTCAGGTCCCAATTC
CXCL10	GGTGAGAAGAGATGTCTGAATCC	GTCCATCCTTGGAAGCACTGCA
ISG56	CAGCAACCATGAGTACAAAT	AAGTGACATCTCAATTGCTC
SeV	GAAAGAGATACCGAACCCAGAG	GCTTGAGGGAGTGTATTGTAGG
