# Supplementary material for: TRIM25 predominately associates with anti-viral stress granules
Source: Nat Commun. 2024 May 15;15:4127. doi: 10.1038/s41467-024-48596-4 (PMC11096359; doi:10.1038/s41467-024-48596-4)
Supplement: Supplementary file 2 — Description of Additional Supplementary Files [file 41467_2024_48596_MOESM2_ESM.pdf]

Description of Additional Supplementary Files

File Name: Supplementary Data 1

Description: A list of G3BP1, TRIM25 and TRIM25ΔPTFG proximity interacting proteins before and after Poly(I:C) treatment was identified by mass spectrometry. HEK293T cells were transfected with G3BP1-BirA*, TRIM25-BirA* and TRIM25ΔPTFG-BirA*. Protein components spatially localized to G3BP1 and TRIM25 were identified by mass spectrometry. Also included are our manually annotated 'Antiviral proteins'.
