# Supplementary material for: TRIM25 predominately associates with anti-viral stress granules
Source: Nat Commun. 2024 May 15;15:4127. doi: 10.1038/s41467-024-48596-4 (PMC11096359; doi:10.1038/s41467-024-48596-4)
Supplement: Supplementary file 3 — Reporting Summary [file 41467_2024_48596_MOESM3_ESM.pdf]

nature portfolio

Corresponding author(s): Da Jia; Peng Bai

Last updated by author(s): May 7, 2024

Reporting Summary

Nature Portfolio wishes to improve the reproducibility of the work that we publish. This form provides structure for consistency and transparency in reporting. For further information on Nature Portfolio policies, see our Editorial Policies and the Editorial Policy Checklist.

Statistics

For all statistical analyses, confirm that the following items are present in the figure legend, table legend, main text, or Methods section.

n/a	Confirmed	
☐	☒	The exact sample size (*n*) for each experimental group/condition, given as a discrete number and unit of measurement
☐	☒	A statement on whether measurements were taken from distinct samples or whether the same sample was measured repeatedly
☐	☒	The statistical test(s) used AND whether they are one- or two-sided<br>*Only common tests should be described solely by name; describe more complex techniques in the Methods section.*
☒	☐	A description of all covariates tested
☒	☐	A description of any assumptions or corrections, such as tests of normality and adjustment for multiple comparisons
☐	☒	A full description of the statistical parameters including central tendency (e.g. means) or other basic estimates (e.g. regression coefficient) AND variation (e.g. standard deviation) or associated estimates of uncertainty (e.g. confidence intervals)
☐	☒	For null hypothesis testing, the test statistic (e.g. *F*, *t*, *r*) with confidence intervals, effect sizes, degrees of freedom and *P* value noted<br>*Give P values as exact values whenever suitable.*
☒	☐	For Bayesian analysis, information on the choice of priors and Markov chain Monte Carlo settings
☒	☐	For hierarchical and complex designs, identification of the appropriate level for tests and full reporting of outcomes
☒	☐	Estimates of effect sizes (e.g. Cohen's *d*, Pearson's *r*), indicating how they were calculated

Our web collection on statistics for biologists contains articles on many of the points above.

Software and code

Policy information about availability of computer code

Data collection	There is no previously unreported custom computer code or algorithm used in this manuscript.<br>SAGECREATION was used to acquire western blot images.<br>Olympus FV3000 was used for confocal imaging of cell lines, and Olympus SpinSR10 was used for live-cell imaging experiments.<br>MS : Q Exactive Plus for identification of G3BP1-proximity labeling proteins and TRIM25-proximity labeling proteins;
Data analysis	Statistical analysis: GraphPad Prism 8; Image quantification: ImageJ and OlyVIA (cell lines; LLPS experiments in vitro and in vivo); MS: MaxQuant version 1.6.2.3

For manuscripts utilizing custom algorithms or software that are central to the research but not yet described in published literature, software must be made available to editors and reviewers. We strongly encourage code deposition in a community repository (e.g. GitHub). See the Nature Portfolio guidelines for submitting code & software for further information.

Data

Policy information about availability of data

All manuscripts must include a data availability statement. This statement should provide the following information, where applicable:

- Accession codes, unique identifiers, or web links for publicly available datasets
- A description of any restrictions on data availability
- For clinical datasets or third party data, please ensure that the statement adheres to our policy

The source data underlying Figs.1/4 are provided as a Source Data file. The mass spectrometry raw data were deposited to the ProteomeXchange Consortium (http://proteomecentral.proteomexchange.org) via the iProx partner repository,with data set identifier IPX0006705000 (Fig1) and IPX0006706000 (Fig3). All the other data supporting the findings of this study are available within the article and its supplementary information files and from the corresponding author upon reasonable request.

Research involving human participants, their data, or biological material

Policy information about studies with human participants or human data. See also policy information about sex, gender (identity/presentation), and sexual orientation and race, ethnicity and racism.

Reporting on sex and gender	n/a
Reporting on race, ethnicity, or other socially relevant groupings	n/a
Population characteristics	n/a
Recruitment	n/a
Ethics oversight	n/a

Note that full information on the approval of the study protocol must also be provided in the manuscript.

Field-specific reporting

Please select the one below that is the best fit for your research. If you are not sure, read the appropriate sections before making your selection.

[x] Life sciences [ ] Behavioural & social sciences [ ] Ecological, evolutionary & environmental sciences

For a reference copy of the document with all sections, see nature.com/documents/nr-reporting-summary-flat.pdf

Life sciences study design

All studies must disclose on these points even when the disclosure is negative.

Sample size	Sample size calculations were not preformed. All experiments were performed using at least 3 independent biological repeats.
Data exclusions	No data was excluded from the analysis.
Replication	All experiments were performed using at least 3 independent biological repeats.
Randomization	Experimental groups were defined based on appropriate biological and technical controls.
Blinding	Not used in this study.

Reporting for specific materials, systems and methods

We require information from authors about some types of materials, experimental systems and methods used in many studies. Here, indicate whether each material, system or method listed is relevant to your study. If you are not sure if a list item applies to your research, read the appropriate section before selecting a response.

Materials & experimental systems

n/a	Involved in the study
☐	☒ Antibodies
☐	☒ Eukaryotic cell lines
☒	☐ Palaeontology and archaeology
☒	☐ Animals and other organisms
☒	☐ Clinical data
☒	☐ Dual use research of concern
☒	☐ Plants

Methods

n/a	Involved in the study
☒	☐ ChIP-seq
☒	☐ Flow cytometry
☒	☐ MRI-based neuroimaging

Antibodies

Antibodies used	Antibodies used in this study were obtained from commercial resources: mouse monoclonal anti-G3BP1 (Proteintech, 66486-1-lg, western blot (WB) 1:1,000, IF 1:300), rabbit anti-G3BP2 (Proteintech, 16276-1-AP, WB 1:1,000, IF 1:300), rabbit anti-TRIM25 (Proteintech, 12573-1-AP, WB 1:1,000, IF 1:300), rabbit anti-TRIM25 (Abcam, ab167154, IF 1:500), rabbit anti-IRF3 (Proteintech, 11312-1-AP, WB 1:1,000), rabbit anti-phospho-IRF3-Ser396, (Abbkine, ABP54922, WB 1:1,000), rabbit anti-IGF2BP2 (Proteintech, 11601-1-AP, WB 1:1,000, IF 1:300), mouse anti-ZC3HAV1 (Proteintech, 66413-1-lg, WB 1:2,000, IF 1:500), mouse anti-PABPC1 (Proteintech, 66809-1-Ig, WB 1:2,000, IF 1:200), mouse anti-RIG-I (Proteintech, 67556-1-lg, WB 1:2,000, IF 1:500), rabbit anti-GFP (Proteintech, 50430-2-AP, WB 1:2,000), rabbit anti-mCherry (Proteintech, 26765-1-AP, WB 1:1,000), rabbit anti-HA, (Proteintech, 51064-2-AP, WB 1:2,000), rabbit anti-GAPDH (Proteintech, 10494-1-AP, WB 1:2,000), rabbit anti-Flag (Proteintech, 20543-1-AP, WB 1:2,000), rabbit anti-ZCCHC3 (ABclonal, A17235, IF 1:200), rabbit anti-Ub-K63, (abmart, 334485, IF 1:300), mouse anti-Ub, (GeneTex, GT7811, WB 1:3,000). Rabbit anti-TIA (abcam, ab140595, IF 1:200), Alexa-labelled secondary antibodies were from Invitrogen (1:2000).
Validation	Antibodies validation was either through the manufacture's validation sheet (see detailed information above for the precise manufacture and ID) or published validation by other research groups (see references list associated with the manuscript).

Eukaryotic cell lines

Policy information about cell lines and Sex and Gender in Research

Cell line source(s)	HEK293T, HepG2, U2OS and HeLa were obtained from ATCC.
Authentication	All cell lines were authenticated by STR profiling.
Mycoplasma contamination	All cell lines were routinely verified to be free of mycoplasma contamination.
Commonly misidentified lines (See ICLAC register)	No commonly misidentified cells were used.
